# Sustainable Methodologies for Efficient Gel Electrophoresis and Streamlined Screening of Difficult Plasmids

**DOI:** 10.3390/mps6020025

**Published:** 2023-03-06

**Authors:** Nadeem Asad, Emily Smith, Sudeep Shakya, Sutton Stegman, Lisa Timmons

**Affiliations:** 1 Department of Molecular Biosciences, The University of Kansas, 1200 Sunnyside Avenue, Lawrence, KS 66045, USA; 2 Department of Biochemistry and Molecular Medicine, 64 Medical Center Drive, University of West Virginia Health Science Center, Morgantown, WV 26506, USA; 3 Department of Neurobiology, Physiology & Behavior, University of California, Davis, CA 95618, USA; 4 Department of Psychiatry and Behavioral Sciences, University of California, Davis, CA 95618, USA; 5 Center for Neuroscience, University of California, Davis, CA 95618, USA

**Keywords:** electrophoresis, agarose, ethidium bromide, sustainability

## Abstract

We describe a workflow for efficient, environmentally attentive, and sustainable practices related to routine agarose gel electrophoresis. The methods reduce plastic waste and improve efficiency, especially for the exhaustive screening of difficult-to-obtain plasmids. Sustainability is increased when agarose is used ten times over by virtue of a thorough recycling regimen. The workflow optimizes workspaces and standardizes lab practices for handling potentially hazardous waste, minimizing environmental harm. Safety, efficiency, and sustainability improve laboratory productivity, help minimize environmental contamination, and increase cost-effectiveness.

## 1. Introduction 

Agarose gel electrophoresis is a basic and necessary technique in many labs. Production and demand for agarose continues to increase due to a rise in electrophoresis applications in industry and medicine, increased use of agarose beads in vaccine production and protein purifications, and a rise in agar usage in the food and agricultural industries. For labs that perform high-volume screening of DNA samples, the cost of agarose can be prohibitive. Bottlenecks in agarose shipping and supply can add to costs and delay experiments, and human activities threaten the health of both farmed and wild algal sources used for agar and agarose production. Indeed, several rounds of laboratory shortages have been reported in recent years due to algal bed collapses, environmental catastrophes such as oil spills, and competition from food-makers [1,2]. A practice of recycling agarose would benefit individual labs in terms of reducing costs, would provide a buffer against supply chain bottlenecks, and, if more widely applied, would be a useful resource management strategy. The workflow for recycling agarose we describe below is efficient, requires limited hands-on effort, and allows agarose to be used at least ten times for gel electrophoresis with no loss of resolution.

Agarose gel electrophoresis is heavily utilized in PCR-based genotyping analyses and in plasmid production and screening. Plasmids are indispensable in molecular biology, with new configurations of DNA required for CRISPR projects, protein production, gene expression analysis, and other forms of molecular exploration. The number of methods described for plasmid production–in excess of 37 (restriction enzyme cloning, ligation independent cloning, Gibson assembly, SLiCE, etc.)–is testament to the value of plasmids in molecular biology [3]. However, even though the evolution of cloning methodologies has focused on efficient plasmid cloning, the fact remains that some plasmids are intrinsically difficult to produce [3]. Difficulties may be incurred due to the large size of the DNA fragments used, high GC content, repetitive sequences, large final size of plasmid, or unexpected technical difficulties and unrealized contaminants [4,5,6,7]. Changing cloning approaches not only incurs additional costs and time, but it is also not guaranteed to overcome cloning hurdles. Another approach to isolating difficult plasmids is to obtain as many colonies as possible using multiple transformation methods (calcium competent and electrocompetent cells), with multiple bacterial strains, and to exhaustively screen all colonies obtained. However, plasmid screening at this scale is time- and labor-intensive. To reduce the costs, time, and labor of screening, we use a Lyse&Load strategy described below which requires little hands-on effort, is limited only by the number of wells available on a gel and minimizes plastic waste. 

The effort involved in plasmid screening has long been a motivating factor for new plasmid screening strategies. Blue-white selection of candidate colonies based on alpha complementation of LacZ fragments [8,9] is simple and effective, but not all vectors or bacterial hosts have the required LacZ gene components. Colony hybridization is an older strategy that effectively screens all colonies on multiple plates, but the protocol is lengthy, requiring days to complete [10,11]. Most often, plasmids are screened for correctness by preparing DNA from candidate colonies and observing restriction enzyme digestion patterns. This incurs cost from enzymes, waste from plastic consumables, and additional time for the DNA preparations and enzyme reactions [12,13]. Much of this work can be avoided by directly screening colonies using PCR or Sanger sequencing [14,15], but this approach also involves expense and time of enzyme reactions. An approach similar to the Lyse&Load strategy described here involves the direct loading of plasmid-containing bacterial colonies into a well of an electrophoresis gel after exposure to denaturing solutions [16]. However, we find that the strategies based on direct analysis of colonies picked from plates can present challenges, especially for newer scientists, such as an inability to re-find or re-grow the original colony, loss of material during gel loading, and insufficient plasmid DNA yield from colonies, especially for larger or lower-copy-number plasmids. These challenges reduce the reliability of directly screening from colonies. The Lyse&Load strategy has proven reliable for us and easy to implement by new trainees.

Our workflow also includes strategies to expedite the production of gels from recycled agarose and efficient ethidium waste disposal. Many labs routinely use ethidium bromide as gel stains, with institutional waste disposal safety requirements that must be met. The workflow we describe maximizes productivity while reducing costs and improves ergonomics related to hazardous waste removal.

## 2. Materials, Solutions, and Methods

**Figure 1:**
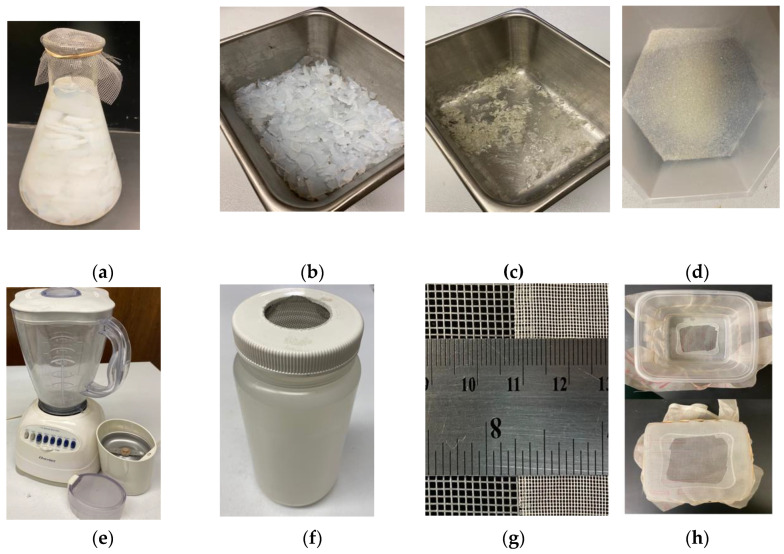
Processing of used agarose gels. (**a**) An Erlenmeyer flask with netting attached to the top allows for efficient removal of water from used gel slices. Gel slices are soaked in distilled water, then with ultrapure (Millipore, filtered) water, with one or more exchanges of water daily. (**b**) After freezing, chopping the gel into finer pieces, and another round of water leaching, gel slices are placed in a baking dish and baked at 55–60 °C until dehydrated; (**c**) depicts dried agarose flakes ready for grinding or storage; (**d**) agarose granules, the final product, after grinding and sifting; (**e**) blender and coffee/spice grinder used in processing; (**f**) depicts the freezer bottles, with lid configured for drainage of thawed pieces; (**g**) displays the relative sizes of the gray polyester mesh (from window screen replacement) used as a strainer for the Erlenmeyer flasks at the Diffusion Stations and after freezing (left). On the right is the bolting fabric used as the sieve for the final product; (**h**) depicts our lab-made sifters made from mesh fabric and a lightweight plastic box. At the top, looking down into the plastic box shows the size of the cutout in the bottom of the box. At the bottom, looking underneath the box shows that the bottom of the box is surrounded by mesh fabric.

**Figure 2:**
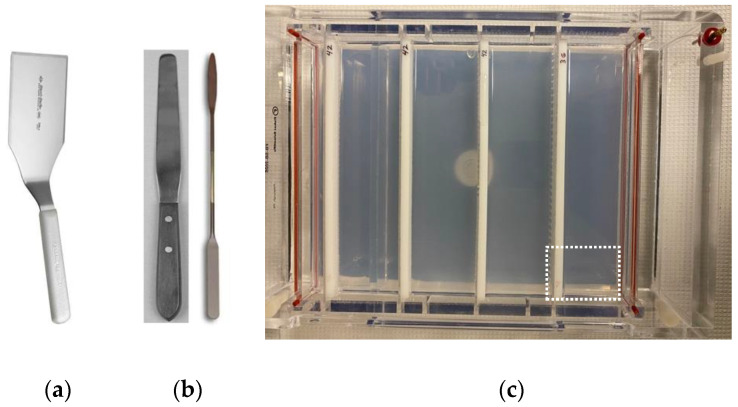
Convenience Gels. (**a**) Because “spatulas” have different shapes, depending on their application, a photo of a spatula is provided that is well-suited for slicing an appropriately sized gel from the Convenience Gel and transferring it to a working gel box; (**b**) a smaller spatula may also be required to help lift the excised gel piece from the Convenience Gel casting box; (**c**) a “Convenience Gel” with 4 rows of combs. Squares are cut from the Convenience Gel, as indicated by white dotted lines. Gel squares are transferred to a new gel box and used for gel electrophoresis.

**Figure 3:**
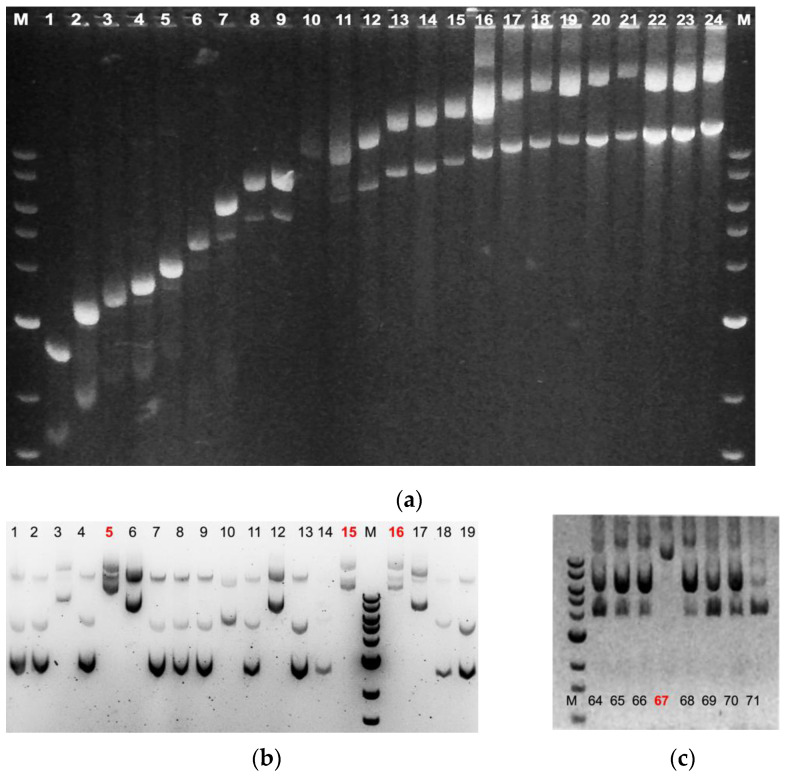
Ability to discriminate supercoiled, undigested plasmids by size using Lyse&Load. The bright RNA bands in these gels are cropped out of the final image or were allowed to run off the gel. Interestingly, the bacterial genomic DNA typically floated out of the well soon after loading. Positive supercoiled DNA runs faster than linearized; relaxed circular DNA runs slower; the supercoiled forms observed depend on the culture conditions. (**a**) A total of 24 different plasmids were selected based on size (see Table 1), cultures were inoculated from frozen bacterial stocks harboring plasmids, and undigested plasmids were loaded onto the gel in order of increasing size on a 1.0% agarose/TAE gel. Depending on the overall lengths of the plasmid, a 5% difference in size can easily be detected. (**b**) Successful screening for a ~12kb plasmid using Lyse&Load. Colonies from a transformation plate were inoculated in 500 μL culture media and shaken at 37 °C for 4–6 h. The culture tubes were centrifuged, and concentrated cells were subjected to Lyse&Load as described. Additional media was added to culture tubes #3, 5, 6, 10, 12,15, 16, and 17 for overnight culture and molecular analysis the following day. Plasmids #5, #15, and #16 were correct. The cloning strategy used here gave rise to multiple classes of plasmids with incorrect (smaller) inserts than expected. (**c**) Lyse&Load screening for a 13,278 bp plasmid. The cloning strategy did produce the correct product, but at a low frequency. The correct version was identified through brute force screening of colonies produced using several different transformation strategies. One correct plasmid (#67) out of >100 colonies screened was found. (M = molecular weight marker). The MW marker sizes are: 12, 10, 8, 6, 4, 3, 2, 1.5, 1, in kilobases.

**Figure 4:**
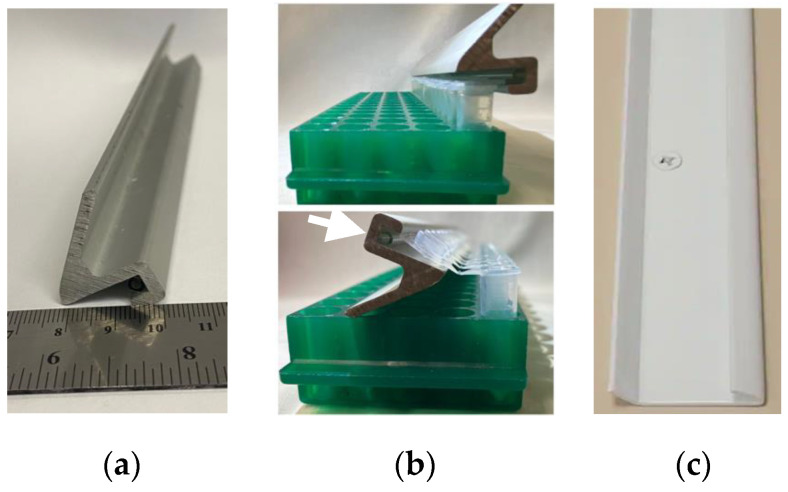
Example of a multi-microtube opener. (**a**) The narrow U-shape accommodates the lid of microtubes, and the metal is sturdy enough to serve as a lever. (**b**) At top, a row of closed microtubes is positioned for opening. At bottom, tubes are opened by twisting the metal in a circular motion over the top of the tubes. The white arrow highlights the 1 mL plastic pipette that was glued into this metal shape to make a narrower groove and better accommodate the microtube lids. (**c**) A product, used for shelving and hanging, that can be purchased inexpensively for this purpose.

**A.**  **Diffusion Stations and grinding methods for recycling agarose gels used in electrophoresis.** An agarose gel can be used more than once in gel electrophoresis, saving cost and time [17]. Processing used agarose gels before reuse, by allowing ethidium bromide, DNA, and salts to diffuse from the gel, gives superior performance compared to simply remelting a used agarose gel [18,19]. We optimized the efficiency of recycling by setting up dedicated stations and materials for this purpose. The steps involve water changes, freezing gel fragments, and draining [20]. The cleaned gel pieces are baked to dryness and ground into powder. Batches of dried gel chips can be stockpiled, reducing the number of times per year that grinding is performed.
  **i.**  **Materials**
    ▪ House-distilled water and Ultrapure (filtered or distilled) water.
    ▪ Two large (2 L) Erlenmeyer flasks set up as Diffusion Stations. These will hold slices of used agarose gel (Figure 1a). One is filled with house-distilled water and placed next to the Gel Documentation System. These are covered by a square of coarse mesh, held in place by a rubber band (Figure 1a).
    ▪ Three stainless or glass baking dishes (Figure 1b,c) (The dried agarose adheres less to stainless material than glass.)
    ▪ Coarse-grade flexible plastic mesh or netting (Figure 1g), with hole size ~1 mm^2^, is derived from polyester window screen replacement material. This is used to cap the Erlenmeyer flasks (Figure 1a) and plastic bottles for freezing gel slices (Figure 1f), yet allow for removal of water.
    ▪ A second, finer mesh is derived from a silk bolting cloth for flour milling (Figure 1g,h). Milling used in silk screening should also work. Other sources of mesh screen that can be inexpensively obtained might include nylon tulle fabric, butterfly netting material, aquarium fish netting, or some nettings used in vegetable or fruit shipments. Gauze and water-absorptive fabrics would not be suitable. It would be good to have extra material (~3 m^2^) on hand to replace damaged squares.
    ▪ Rubber bands.
    ▪ Plastic or metal kitchen spatulas (Figure 2a).
    ▪ Plastic box for sifting (15 cm^2^ and walls 10 cm high or larger), thin-walled. A hole is cut into the bottom with scissors or a safety razor, and mesh is secured with a rubber band (Figure 1h) or glued using cyanoacrylate adhesive (Superglue).
    ▪ Large plastic bottles with wide lids that are freezer-safe. These will be used to freeze, store, and drain agarose pieces. Cut a wide hole into the lid (we used a woodworking hole saw for this purpose) and cover the hole from the underside with a section of the coarser mesh. Glue the mesh onto the lid (Figure 1f) using cyanoacrylate adhesive (Superglue).
    ▪ Wide funnel with large opening (optional).
    ▪ Incubator or drying oven, with fan, set at 55–60 °C.
    ▪ Coffee/spice grinder or food blender (Figure 1e).
    ▪ Large (6 L+) plastic pail or tub for storing dried agarose chunks.
    ▪ Wide-mouth containers, with lid, for storing the final recycled agarose product.
    ▪ Protective gloves and bench-protecting absorbent paper.
    ▪ Decontamination Vessel for used buffer with ethidium (Section C).
  **ii.**  **Methods**
    ▪ The steps below are performed in an area restricted to ethidium bromide use while wearing gloves, and all materials are confined to the area. Gels and gel pieces are moved with spatulas reserved for this purpose and are not directly handled.
    ▪ After photo documentation of the results from agarose gel electrophoresis, a spatula is used to break apart the gel into several narrow, rectangular strips. The gel can be fragmented on the UV transillumination box directly using a plastic spatula. The strips are placed into an Erlenmeyer flask positioned at the 1st Diffusion Station near the gel documentation system.
    ▪ The gel pieces in the 1st Diffusion Station are covered with distilled water. The distilled water is replaced with fresh water at least once per day. The mesh is attached to the Erlenmeyer (Figure 1a) and the water is emptied into the Decontamination Vessel (Section C) for removal of ethidium bromide.
    ▪ When the Erlenmeyer flask in the 1st Diffusion Station is full of gel fragments, it is then moved to the 2nd Diffusion Station, where it is filled with Ultrapure water (Millipore filtration system). A new Erlenmeyer is then placed at the 1st Diffusion Station; thus, two Erlenmeyers are in service at two Diffusion Stations.
    ▪ The water from the Erlenmeyer in the 2nd Diffusion Station is replaced daily for ~3 days by emptying into the Decontamination Vessel (Section C).
    ▪ The drained agarose is placed into a baking dish and chopped into finer pieces using a kitchen spatula, soaked in Millipore water for several hours—overnight, and then placed into a large freezer-safe plastic bottle, capped, drained, and frozen at −20 °C (Figure 1f). Frozen gel fragments can be stored for later processing.
    ▪ The agarose is thawed, and the liquid drained by inverting the plastic bottle (Figure 1f) on a funnel in the Decontamination Vessel. Ultrapure water is added to the bottle, and the pieces drained after an overnight soak.
    ▪ The thoroughly drained pieces are placed in a clean baking dish (Figure 1b). The dish is placed in the drying oven set at 55 °C. The gel pieces are completely dried within 24–36 h (Figure 1c).
    ▪ Batches of dried agarose pieces are collected into a large plastic pail, with lid, until they can be ground into powder.
    ▪ The grinding and sifting steps below are performed in a fume hood while wearing a disposable mask. All items are stored in a sealed box until needed.
    ▪ The dried gel pieces can be ground using a kitchen blender, bead beater, or spice/coffee grinder (Figure 1e). A larger kitchen blender allows for hands-free grinding of larger quantities.
    ▪ The ground gel pieces are sifted through the finer mesh fabric. The sieving fabric is secured around a plastic box with a hole cut from the bottom (Figure 1h). The sifted particles are collected in a clean baking dish. Larger particles are homogenized again or discarded. The final product is depicted in Figure 1d).
**B.**  **Recycled gel preparation.** Recycled agarose is used the same as fresh agarose.
  **i.**  **Materials and Methods**
    ▪ For each 100 mL of gel required, weigh 1g of recycled agarose in a bottle, add 100 mL of 1X running buffer, add stir bar, loosely cap and slowly heat using a heated stir plate. The melting point of agarose is 91–95 °C [21], so boiling is not required. When dissolved, let cool to 55–60 °C and pour into gel box. (Cooling avoids heating of the plastic gel box, which, in the long term, could cause warping.) When ethidium bromide is used to detect DNA, it can be incorporated into the protocol at several points. Some labs add ethidium to the gel before pouring [22], some labs add ethidium to both gel and running buffer; other labs perform staining of the gel after running by placing the gel in an ethidium bath [23,24]. In all cases, measures for the safe handling of ethidium and its disposal (especially for ethidium baths) must be devised with the work area and experience level of users in mind.
    ▪ The agarose can be recycled more than ten times. For resolution of small bands (<200 bp) that require 3–4% agarose, we use mixtures of recycled and fresh agarose, depending on the % difference in band sizes we are analyzing.
    ▪ The end life of a batch of recycled gel can be determined by comparing the background fluorescence in a recycled gel to a fresh gel (Results and Discussion0. Agarose that is no longer recyclable can be incinerated along with spent charcoal “tea bags” (Section C).
    ▪ By grinding large batches more infrequently and using one batch of used agarose at a time, it is easier to keep track of how many times a particular lot of used agarose has been recycled.
**C.**  **Decontamination Vessel and strategy for ethidium bromide waste removal.** For ethidium bromide users, proper disposal can be inconvenient, but better for the environment and required by institutes. Setting up efficient methodology, a dedicated work area, and dedicated containers is key to managing this process efficiently.
  **i.**  **Materials**
    ▪ Carboy with working spigot and wide opening to allow for neat pouring of water from the Erlenmeyer flasks used at Diffusion Stations for agarose recycling. The carboy is the Decontamination Vessel for the removal of ethidium bromide from agarose gel washes and from running buffer. The spigot feature of the carboy allows for quick discarding of decontaminated liquid. A plastic bottle can also be used.
    ▪ Large funnel with wide opening.
    ▪ Large 100 cc syringe for transferring ethidium-containing liquid or buffer to Decontamination Vessel.
    ▪ Activated charcoal “tea bags” purchased from scientific supplier. For example: Biotium Destaining Bags/VWR (supplier #22007, catalog #89427-060) or MP Biomedicals^®^ (SKU #112350200, CAS Number #: 7440-44-0). One bag can remove 10 mg of ethidium bromide from solutions, with 95% removed in 4 h (product MSDS data sheets). Alternatively, bags can be made from activated charcoal (from aquarium or lab suppliers) wrapped with mesh and secured with a rubber band.
    ▪ Stir-only stir plate (no heat) and large stir bar.
    ▪ Twine. The charcoal bag should remain in solution near, but not touching, the bottom of the Decontamination Vessel, and the twine should extend from the bag to the outside of the carboy so that the bag can be removed without users contacting the ethidium-containing solution.
    ▪ Incinerator, for municipalities that do not incinerate trash (see your Environmental Safety or Animal Care unit).
  **ii.**  **Methods**
    ▪ Used electrophoresis buffer containing ethidium bromide is transferred from the gel box to a dedicated Decontamination Vessel. A small length of tubing attached to a large (100 cc) syringe, dedicated for this purpose, is a safe and convenient strategy for liquid transfer. If ethidium is incorporated into the agarose gel, but not the running buffer, the running buffer will contain ethidium at the end of a run, especially at the anode, as ethidium is positively charged.
    ▪ The Decontamination Vessel can be placed on a stir plate (incapable of heating, for safety reasons) for more efficient adsorption of ethidium by the charcoal bags. Attaching twine to the “tea bag” would help ensure that the bag is easily retrievable.
    ▪ It is convenient to close the Decontamination Vessel to liquid collection on a specific day (Friday evening), then empty the Vessel 24–48 h later (Monday morning). According to MSDS, a decontamination time of 4 h is sufficient for removal of 95% of ethidium bromide.
    ▪ Keep track of how many liters of original agarose gel buffer (and therefore how many mg of ethidium bromide) have been adsorbed by the “tea bag” so that its binding capacity is not exceeded.
    ▪ When spent (10 mg capacity), the “tea bag” can be placed in a sealed plastic bag and stored until incineration. Ethidium bromide decomposes at 262 °C, so autoclaving is not sufficient for inactivation.
**D.**  **Convenience Gels for time-efficient agarose gel production.** The fact that agarose gels can be prepared in advance and stored for weeks before use is not commonly appreciated. The EDTA in TAE and TBE buffers helps prevent growth of organisms. Dehydration and diffusion of ethidium bromide from gels can be prevented by keeping the gels submerged in 1X TAE (or TBE) Running Buffer with ethidium bromide added. Pre-casting “Convenience Gels” saves time, as users do not have to wait for heated agarose to dissolve, cool, and set, and more gel boxes are available for running gels as they are not being used for casting.
  **i.**  **Materials**
    ▪ Large agarose gel casting tray with lid (the 20 cm wide × 27 cm long casting tray from Fisher scientific in Figure 2 remains in the gel box during casting). The box is used for gel casting and storage and need not be functional.
    ▪ Large gel combs that fit the casting tray (for example, 4 combs, with 36 or 42 wells).
    ▪ Agarose, electrophoresis buffer, and heat source for melting.
    ▪ Running buffer (1X TAE or 1X TBE).
    ▪ Plastic or stainless kitchen spatula. A narrow, rectangular shape is better than flared. Squared edges work better than rounded edges (Figure 2a).
  **ii.**  **Methods**
    ▪ Determine the amount and percentage of agarose needed, melt agarose in running buffer, cool and pour gel into casting tray.
    ▪ Use combs to create a 3- or 4-tiered set of mini-gels (Figure 2c).
    ▪ When gel is set, cover the gel with running buffer to prevent the gel from drying out. If the gel contains ethidium bromide, then the equivalent concentration of ethidium bromide should be added to the running buffer to prevent diffusion of the stain from the gel. Running buffers with ethidium should be placed in the Decontamination Vessel when finished. If watertight, the gel dams can be left in place to conserve use of the running buffer. Place lid on gel box to protect from dust and insects.
    ▪ The Convenience Gels can be stored on the bench for 2 weeks or more without loss of resolution. The gels should be always covered with running buffer.
    ▪ When a gel is needed, a section of the Convenience Gel with the required number of intact wells is excised using a spatula (Figure 2a) and carefully transferred to a new gel box. Wells on the ends are sacrificed in the cutting because the gel cannot be sliced cleanly between wells without loss of gel integrity. When cutting in the middle of a well, the neighboring well is not usually damaged.
**E.**  **Lyse&Load Protocol for rapid screening of plasmids based on size**. When making a new plasmid, individual colonies must be screened to ensure that the desired plasmid is obtained and configured as designed. With this Lyse&Load protocol, it is possible to screen colonies, based on the relative size of the plasmid. This procedure uses solutions from the original alkaline lysis protocol [13] and is more reproducible than the single colony lysate protocol [16] through the addition of a step to scale up bacterial cell numbers. Moreover, the methodology is designed to minimize waste and hands-on time involved in screening.
      The goal is to take a small amount of concentrated bacterial cell suspension and quickly load the cell contents onto a gel for size analysis of uncut plasmids. If positive results are obtained for a particular culture tube, the remainder of the cell pellet is diluted with fresh growth media and cultured overnight for miniprep production the next day, analyzed by PCR, or other form of downstream analysis. Thus, DNA is isolated and further analyzed only from colonies that have a good chance of being correct. As further analysis involves use of restriction enzymes, PCR, and/or DNA sequencing, costs and effort are drastically reduced when screening at a large scale.
  **i.**  **Materials**
    ▪ Sterile tubes for bacterial culture and appropriate culture media. Glass culture tubes with metal caps are reusable and help minimize plastic waste.
    ▪ Swinging bucket centrifuge that can accommodate the bacterial culture tubes (for example: Beckman Coulter Allegra X-30R or Eppendorf 5810).
    ▪ Inoculation loop and flame.
    ▪ Liquid aspirator.
    ▪ Vortex Mixer.
    ▪ Parafilm sheets.
    ▪ Sterile pipet tips.
    ▪ Agarose gel electrophoresis system.
    ▪ Gel documentation system.
    ▪ Solutions:
      ○ 3X Alkaline Lysis Solution:
        3% SDS,
        0.6 M NaOH in water
        (This solution can be stored at room temperature for at least 2 months, but may need heating to ensure SDS is dissolved.)
      ○ Loading dye (other dyes can be used; store frozen):
        60% glycerol,
        1X TAE (or preferred running buffer),
        0.3% Bromophenol Blue
      ○ Lyse&Load Solution: (make fresh)
        9 parts 3X Alkaline Lysis Solution
        1 part Loading Dye
        (10 μL will be needed for each sample.)
  **ii.**  **Materials and Methods**
    ▪ Using a sterile, flamed inoculation loop, inoculate colonies in culture tubes and incubate with shaking at 37 °C. For rapid, same-day screening, use a small volume of culture media (250–500 μL). For slow-growing colonies or low-yield plasmids, it may not be possible to obtain results in one day and an overnight culture would be required. For overnight culture, increase the volume of culture media as usual.
    ▪ When the culture media is turbid (~OD_595_= 0.6–0.9), or after overnight culture, place the tubes in a swinging bucket centrifuge, balance, and obtain a cell pellet by spinning ~3000 rpm for 5–15 min (higher centrifugal forces may stress glass tubes.)
    ▪ Most of the supernatant is removed from the cell pellet and discarded into a decontamination vessel, leaving behind ~10% of the original volume of media. The glass tubes are then vortexed in the remaining growth media to resuspend the cell pellet to generate a more concentrated suspension of cells.
    ▪ Prepare a 1% agarose gel, or using Convenience Gels, cut out the number of lanes you will need, and fill the gel box with running buffer.
    ▪ For quick work, a large square of parafilm is pressed onto the work surface in the gel area and the protective paper backing is removed. The parafilm will hold spots of liquid, taking the place of microfuge tubes. This reduces plastic waste and saves time.
    ▪ Spot 10 μL of Lyse&Load Solution onto the parafilm sheet; one spot for each concentrated cell suspension you will analyze. Make sure to leave space between each dot so they will not merge when the sample is added to them.
    ▪ Load the Molecular Weight marker on the gel. Another good control to use for sizing is uncut plasmid vector used in the construction of the plasmid.
    ▪ Into each 10 μL spot of Lyse&Load Solution, add 20 μL of concentrated cell pellet. Carefully pipette up and down to mix, avoiding bubbles, and *immediately* load 20 μL of the mixture onto the gel.
    ▪ Samples are mixed and loaded onto the gel one at a time. With delays in loading, the now-lysed bacterial contents will stick to the pipette tip, and the contents will float out of the well.
    ▪ Depending on the loading dye used, the high pH of the Lyse&Load solution may alter the color of the dye, making it difficult to see. Wells loaded with lysed cells typically have an opaque appearance. A dark colored sheet of paper underneath the gel provides contrast and helps with keeping track of loaded wells.
    ▪ Run gel at appropriate voltage for apparatus.
    ▪ Compare relative sizes of plasmids (see Figure 3).
**F.**  A Multi-tube opener for speedy and ergonomic microtube (1.5–2 mL) lid opening. Tube manufactures supply plastic tube openers that can open one microtube at a time. For those with arm or hand injuries, these ergonomic devices are indispensable. We describe a device that can open an entire row of microtubes simultaneously, saving time and strain on hands. When several plasmids are screened simultaneously using Lyse&Load, handling the microtubes is easier with the Multi-tube opener. The opener reduces stress on hands and fingers and saves time.
  **i.**  **Materials and Methods**
    ▪ The device we used (Figure 4a) was a lid support from broken equipment. We cut this metal piece to the length of a 16 × 5-tube rack using a bandsaw. It is an appropriately shaped and sturdy piece of metal and is a good lever for opening plastic microtubes. We investigated purchasable products that could serve the same purpose and found that hang rails/hanging tracks used in picture and shelf hanging will also work. We tested an Everbilt Super Duty Hang Track, 2in (Home Depot #1000 025 203) and found that it can also work for this purpose (Figure 4c).
    ▪ Bandsaw for cutting to size.
    ▪ A 1 mL plastic serological pipette. The groove in our Multi-tube Opener was too deep for the shape of the microtube lid, and this prevented efficient tube opening. We made the groove shallower by using cyanoacrylate adhesive (Superglue) to attach the pipette in the groove (Figure 4b, arrow).
    ▪ The metal bar is used as a lever to open a row of tubes (Figure 4b).

## 3. Results and Discussion

An expansion in agarose gel applications related to biotechnology, medicine, and molecular biology have increased demand for agar and agarose, which is derived from multicellular red algae (*Gracilaria* or *Gracilariopsis*). Agarose is refined from agar by removing agaropectin and is comprised of a linear, neutral polysaccharide with repeated units of the disaccharide agarobiose, a linkage of D-galactose and 3,6-anhydro L-galactose [25]. Most seaweed for agar production is harvested from wild sources, although aquaculture production is increasing, principally in China, Korea, the Phillipines, Japan, and Chile [26,27]. Industries and applications outside laboratory research also require agar and agarose. For example, seaweed is a source of dietary protein and included in health supplements in humans [28,29,30]. As a dietary supplement in ruminants, it can reduce methane production, highlighting an important potential for ameliorating biological carbon emissions [31]. Red algae are also a biomass feedstock for biofuel production [32]. These non-laboratory demands for agarose can contribute to supply chain bottlenecks. Disease, ocean pollution, invasive species, and general decline in the aquatic environment, which have reduced the quantity of wild *Gracilaria/Gracilariopsis* mined in recent years, can also limit the supply of agar and agarose [33]. Shortages have affected laboratories, with agar shortages in 2016 spurring rationing by suppliers; for example, [34]. Recycling agarose can not only reduce costs but also buffer against shortages. 

**Figure 5:**
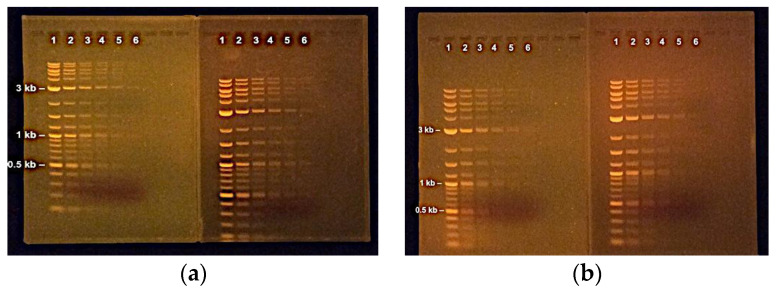
Use of recycled agarose. Pairs of 1% agarose/TAE gels were prepared under identical conditions and were electrophoresed in the same gel box for the same time. For each pair, the gel on the left contains recycled agarose; the gel on the right was made using new agarose. (**a**) The dehydrated gel pieces were sifted using the coarser mesh only. The recycled agarose required ~5min longer to dissolve than the new agarose, due to the presence of larger particles. (**b**) The dehydrated gel pieces were sifted through the finer mesh and required only ~40sec longer to dissolve than the new agarose. The two gels, recycled versus new, ran similarly. Overall, the coarser ground agarose particles provided good resolution, but the DNA migrated slower in comparison to finer used agarose particles or fresh reagent. This highlights an advantage of sifting. The DNA loaded is a 1 in 2 dilution series of Molecular Weight DNA standards (NEB Purple 1Kb ladder, Plus). Band sizes are 10, 8, 6, 5, 4, 3, 2, 1.5, 1.2, 1, 0.9, 0.8, 0.7, 0.6, 0.5, 0.4, 0.3, 0.2, 0.1 in kilobases.

The agarose recycling workflow we describe requires minimal daily effort, and agarose can be recycled ten times with little loss in resolving power. The samples in Figure 5 were loaded onto gels made with fresh or recycled agarose, for comparison. Recycled agarose is suited for most experimental requirements, except in cases where DNA must be excised from gels, when the yield of DNA is anticipated to be low (Figure 5 and Table 1), or when DNA fragments are small (less than 200–300 bp

By setting up Diffusion Stations for agarose recycling as described, water changes can be quickly performed each day with little effort. With two stations employed simultaneously, the used gel can be processed and baked into chips within 7 days. Given the Diffusion Coefficient measured for a 1000 bp DNA fragment in 2% agarose (0.52 × 10^−7^ cm^2^ s^−1^) [35], we estimate the time required for such a DNA fragment to diffuse from a 0.5 cm thick agarose gel slice to be 6.9 days. This calculation does not consider water exchanges, which would speed up the cleaning of agarose chunks. We performed water exchanges at least once per day and the ratio of gel fragment volume to water volume was 1:1 or less. As the Diffusion Coefficient of Ethidium Bromide is over 2000X higher for DNA as measured in aqueous solutions (4.2 × 10^−4^ cm^2^ s^−1^ versus 20 × 10^−8^ cm^2^ s^−1^), the ethidium bromide should be eliminated from the gel pieces during this time frame [36,37].

**Figure 6:**
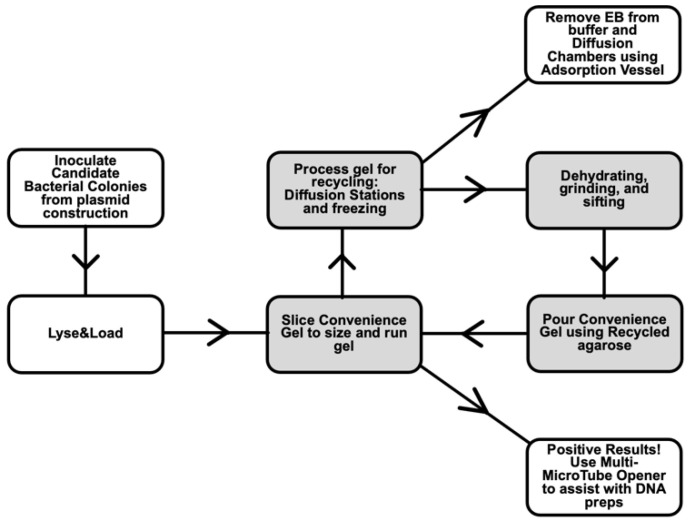
Workflow for difficult plasmid screening and use of recycled agarose. The gel recycling steps, highlighted in gray boxes, are relatively straightforward, requiring only water exchanges, collecting gel pieces, freezing gel pieces, and drying them in an oven. The daily hands-on effort is minimal. More time is required for grinding and sifting, but this only needs to be performed a few times per year, as the dried agarose can be stored until enough is accumulated for processing.

Coupled with agarose recycling, we have created a workflow to help eliminate ethidium bromide from laboratory waste (Figure 6). While the mutagenic capacity of ethidium bromide in humans is not well established [38] and the question of permeability to live cells and tissues still lingers [39], safety offices in most institutions have strict regulations regarding its use and disposal [40]. The effects of ethidium bromide on mitochondrial DNA [41,42] warrant careful use and disposal. Moreover, there are growing concerns regarding the effects of ethidium bromide released into the environment on fish and other wildlife [43]. With a streamlined system for disposal, we find the charcoal absorption method, followed by incineration, to be efficient, inexpensive, and an environmentally responsible means to handle these dilute solutions of ethidium bromide and meet the requirements of institutions’ safety protocols [44]

The workflows that we developed (Figure 6) have significantly improved the speed and efficiency of plasmid screening, allowing hundreds of colonies to be efficiently screened in a short time for those plasmids that prove difficult to produce. Devising a means to pre-cast Convenience Gels and store them for weeks without loss of stain or dehydration adds convenience and helps save time. Using Lyse&Load, one correct plasmid can easily be found in a background of hundreds of false-positive colonies in one day; the number of colonies that can be screened is limited by the number of wells available in gels. Screening by size is possible in cases where the final plasmid is larger (or smaller) than the starting vector. Figure 3a and Table 1 demonstrate that small differences in uncut plasmids can be distinguished (5% or less, depending on plasmid size). Figure 3a,c demonstrate successful implementation of the Lyse&Load protocol, allowing us to isolate plasmids that were difficult to make due to incorporation of extraneous DNA (Figure 3b) or due to the large final size of the plasmid (Figure 3c). 

The “Lyse&Load” modifications to existing plasmid screening protocols that we made, while small and incremental, have made a large impact on our ability to quickly screen plasmids in a reproducible fashion. By increasing the alkaline lysis solution to 3X concentration, by starting with a small, concentrated pool of cells, and by taking care not to expose the cells to lysis solutions for too long before loading on the gel, we have eliminated several frustrations common to colony screening. Our plasmids no longer float out of the wells along with the genomic DNA, and the yields are sufficient for the observation of large plasmids and lower copy number plasmids on the gels. This Lyse&Load protocol is routinely and successfully performed by undergrads in the lab. Moreover, as we use reusable glass culture tubes, the only waste products are pipet tips and a sheet of parafilm. 

The collection of methods we describe, agarose recycling, ethidium waste removal strategies, Convenience Gel preparation, Lyse&Load, and an ergonomic Multi-Microtube opener, can be incorporated into a streamlined workflow (Figure 6) to allow for efficient, safe, and sustainable lab practices. 

## Figures and Tables

**Table 1:** Screening supercoiled, undigested plasmids by size. The size of each plasmid in Figure 3a is listed, along with the differences in absolute length (in base pairs) and % difference in length between the indicated plasmid and the one in the previous well.

Lane	Length (bp)	Δ Length (bp)	% Difference
1	2641		
2	3228	587	22.2
3	3488	260	8.1
4	3630	142	4.1
5	3970	340	9.4
6	4401	431	10.9
7	5236	835	19.0
8	5774	538	10.3
9	6044	270	4.7
10	6986	942	15.6
11	7265	279	4.0
12	7767	502	6.9
13	8796	1029	13.2
14	9225	429	4.9
15	9749	524	5.7
16	11,181	1432	14.7
17	12,147	966	8.6
18	12,695	548	4.5
19	13,278	583	4.6
20	13,652	374	2.8
21	14,162	510	3.7
22	14,971	809	5.7
23	15,479	508	3.4
24	17,577	2098	13.6

## Data Availability

Not applicable.
